# Identification and Signature Sequences of Bacterial Δ^4,5^Hexuronate-2-*O*-Sulfatases

**DOI:** 10.3389/fmicb.2019.00704

**Published:** 2019-04-05

**Authors:** Shumin Wang, Jingwen Guan, Qingdong Zhang, Xiangxue Chen, Fuchuan Li

**Affiliations:** ^1^National Glycoengineering Research Center and Shandong Provincial Key Laboratory of Carbohydrate Chemistry and Glycobiology, Shandong University, Qingdao, China; ^2^Dongying Tiandong Pharmaceutical, Co., Ltd., Dongying, China

**Keywords:** glycosaminoglycan, chondroitin sulfate, dermatan sulfate, sulfatase, signature sequence

## Abstract

Glycosaminoglycan (GAG) sulfatases, which catalyze the hydrolysis of sulfate esters from GAGs, belong to a large and conserved sulfatase family. Bacterial GAG sulfatases are essential in the process of sulfur cycling and are useful for the structural analysis of GAGs. Only a few GAG-specific sulfatases have been studied in detail and reported to date. Herein, the GAG-degrading *Photobacterium* sp. FC615 was isolated from marine sediment, and a novel Δ^4,5^hexuronate-2-*O*-sulfatase (PB2SF) was identified from this bacterium. PB2SF specifically removed 2-*O*-sulfate from the unsaturated hexuronate residue located at the non-reducing end of GAG oligosaccharides produced by GAG lyases. A structural model of PB2SF was constructed through a homology-modeling method. Six conserved amino acids around the active site were chosen for further analysis using site-directed mutagenesis. N113A, K141A, K141H, H143A, H143K, H205A, and H205K mutants exhibited only feeble activity, while the H310A, H310K, and D52A mutants were totally inactive, indicating that these conserved residues, particularly Asp52 and His310, were essential in the catalytic mechanism. Furthermore, bioinformatic analysis revealed that GAG sulfatases with specific degradative properties clustered together in the neighbor-joining phylogenetic tree. Based on this finding, 60 Δ^4,5^hexuronate-2-*O*-sulfatases were predicted in the NCBI protein database, and one with relatively low identity to PB2SF was characterized to confirm our prediction. Moreover, the signature sequences of bacterial Δ^4,5^hexuronate-2-*O*-sulfatases were identified. With the reported signature motifs, the sulfatase sequence of the Δ^4,5^hexuronate-2-*O*-sulfatase family could be simply identified before cloning. Taken together, the results of this study should aid in the identification and further application of novel GAG sulfatases.

## Introduction

Sulfatases, which catalyze the cleavage of sulfate esters, participate in the metabolic pathways of bacteria that utilize sulfated polysaccharides as carbon sources (Hanson et al., 2004). The desulfation of these biopolymers by sulfatases facilitates their degradation by other enzymes and is essential for the bioremediation of organosulfur compounds derived from plants and animals (Kertesz, 2000; Sogi et al., 2013; Barbeyron et al., 2016b). The marine environment is rich in heterogeneously sulfated polysaccharides, such as sulfated glycosaminoglycans (GAGs), carrageenans, and fucans/fucoidans (Berteau and Mulloy, 2003; Pavao, 2014; Vasconcelos and Pomin, 2017). GAGs are linear polyanionic polysaccharides with repeating disaccharide units and are the major compounds of the extracellular matrix of the animal cells. Chondroitin sulfate (CS)/dermatan sulfate (DS) and heparin (Hep)/heparan sulfate (HS) are highly sulfated GAG members. CS/DS polysaccharides are composed of repeating disaccharide units containing hexuronic acid and *N*-acetyl-D-galactosamine (GalNAc). The hexuronic acid in CS is β-D-glucuronic acid (GlcA), while GlcA is converted to α-L-iduronic acid (IdoA) by a C-5 epimerase in DS (Maccarana et al., 2006). Therefore, CS/DS polysaccharide chains usually exist in a hybrid form. In contrast, the disaccharide unit of Hep/HS is composed of hexuronic acid (GlcA/IdoA) and *N*-acetyl-D-glucosamine (GlcNAc) (Carlsson et al., 2008). CS/DS and Hep/HS are further modified by various sulfotransferases to form complex structures with different sulfation patterns (Sugahara and Kitagawa, 2000). Different GAG chains with unique sulfation patterns are involved in various physiological and pathological processes (Bishop et al., 2007), such as brain development, wound repair, and cancer metastasis (Poole, 1986; Afratis et al., 2012; Ghatak et al., 2015). Saccharide chains can be specifically modified via desulfation by sulfatases, making the GAG sulfatases potential tools for the preparation and structure-function analyses of oligosaccharides with unique structures.

Glycosaminoglycan sulfatases belong to a conserved sulfatase family that hydrolyzes specific sulfate esters in saccharide rings (Hanson et al., 2004). Based on the position of the sulfate group selectively removed by sulfatases (Wang et al., 2016), these enzymes can be classified into several types, such as *N*-acetylgalactosamine-4-*O*-sulfatase and *N*-acetylgalactosamine-6-*O*-sulfatase, which specifically hydrolyze sulfate groups on the C-4 and C-6 positions of GalNAc, respectively, and Δ^4,5^hexuronate-2-*O*-sulfatase, which specifically removes C-2 sulfate groups from the unsaturated hexuronic acid derived from the digestion of the β1-4 glycosidic linkage between GlcNAc/GalNAc and GlcA/IdoA units by GAG lyases (Ernst et al., 1995). Based on the sequence similarities, the GAG sulfatases were recently classified in the database SulfAtlas (Barbeyron et al., 2016a). The *N*-acetylglucosamine-6-*O*-sulfatases (EC 3.1.6.14) belong to the family S1 of sulfatases (formylglycine-dependent sulfatases), subfamily 6 (family S1_6; eukaryotic enzymes); The *N*-acetylgalactosamine-6-*O*-sulfatases (EC 3.1.6.4) belong to the family S1_5 (eukaryotic enzymes) and S1_15 (prokaryotic enzymes); The *N*-acetylgalactosamine-4-*O*-sulfatases (EC 3.1.6.12) belong to the family S1_2 (eukaryotic enzymes) and S1_27 (prokaryotic enzymes); The *N*-sulfoglucosamine-6-*O*-sulfatases (EC 3.1.6.11) belong to the family S1_11 (prokaryotic enzymes); The iduronate/glucuronate-2-*O*-sulfatases (EC 3.1.6.13/18) belong to the family S1_7 (eukaryotic enzymes) and the Δ^4,5^hexuronate-2-*O*-sulfatases belong to the family S1_9 (prokaryotic enzymes). Finally, the *N*-sulfoglucosamine-2-*N*-sulfatases (EC 3.10.1.1) belong to the family S1_8 (prokaryotic and eukaryotic enzymes). However, most of the identified GAG sulfatases from animals and bacteria only efficiently act on the sulfate groups at the terminal disaccharides of GAG chains and are therefore referred to exo-sulfatases (Sugahara and Kojima, 1996; Raman et al., 2003; Myette et al., 2009; Malleron et al., 2012). In recent years, several endo-sulfatases discovered in mammals (Dhoot et al., 2001; Morimoto-Tomita et al., 2002) and bacteria (Ulmer et al., 2014; Wang et al., 2015) were found to remove internal sulfate groups from polysaccharides. GAG sulfatases with distinct degradation patterns play a vital role in polysaccharide degradation, during which endo-sulfatases remove sulfate groups from GAGs to facilitate the cleavage of polysaccharide chains by GAG-degrading enzymes (Wang et al., 2015). The resulting oligosaccharides, particularly disaccharides, are further desulfated by exo-sulfatases and subsequently hydrolyzed by glucuronyl hydrolases to produce monosaccharides and sulfates, which may be used as carbon and sulfur sources for cell growth (Ernst et al., 1995; Yamada, 2015).

Arylsulfatases, including GAG-specific sulfatases, possess highly conserved N-terminal Cys/Ser-X-Pro-X-Arg sequences. The conserved Cys/Ser residue is oxidized to an aldehyde, FGly (α-formylglycine), through post-translational modification (Dierks et al., 1998; Boltes et al., 2001). The FGly residue is the critical catalytic residue in the sulfatase catalytic center (Schmidt et al., 1995; Recksiek et al., 1998). To date, only a few bacterial sulfatases acting on CS/DS have been reported, including 4-*O*-sulfatase (S1_27; GenBank^TM^ Accession No. AJK90566) from *Vibrio* sp. FC509 (Wang et al., 2015), 4-*O*-sulfatase BT_3349 (S1_27; AAO78455) from *Bacteroides thetaiotaomicron* VPI-5482, 2-*O*-sulfatase BT_1596 (S1_9; NP_810509) from *Bacteroides thetaiotaomicron* VPI-5482 (Ulmer et al., 2014), 2-*O*-sulfatase Phep_2825 (S1_9; WP_015808635) from *Pedobacter heparinus* DSM 2366 (Myette et al., 2003), and 6-*O*-sulfatase BT_3333 (S1_15; NP_812245) from *Bacteroides thetaiotaomicron* VPI-5482. The rare protein sequences characterized in databases including GenBank and SulfAtlas do not provide enough information for the reliable assignment of GAG sulfatases via bioinformatic analysis. Therefore, although some candidate genes are predicted to be GAG sulfatases, their degradation specificity may be falsely annotated. As GAG sulfatase is an essential enzyme in the degradative metabolism and structural analysis of GAGs, it is important to identify additional types of sulfatases to enrich the sulfatase database.

In this study, a Δ^4,5^hexuronate-2-*O*-sulfatase, designated PB2SF, was identified from the Gram-negative marine bacterium *Photobacterium* sp. FC615, which is the first reported *Photobacterium* that can utilize multiple GAGs. The catalytic mechanism of this enzyme was investigated via homology modeling and site-directed mutagenesis. On this basis, we predicted another 60 Δ^4,5^hexuronate-2-*O*-sulfatases in GenBank using a bioinformatic method, and we report their signature sequences. To confirm our hypothesis, another novel 2-*O*-sulfatase with low identity to PB2SF was also cloned and identified.

## Materials and Methods

### Materials and Bacterial Strains

The strains and plasmids used in this study are listed in Table 1. PrimeSTAR^TM^ HS DNA polymerases, restriction endonuclease and other genetic engineering enzymes were purchased from Takara, Inc. (Dalian, China). A Genome Extraction Kit was purchased from Tiangen Biotech, Co., Ltd. (Beijing, China). Fast Mutagenesis System Kit was purchased from TransGen Biotech, Co., Ltd. (Beijing, China). Standard GAG unsaturated disaccharides were purchased from Iduron (Manchester, United Kingdom). CS-C and CS-D from shark cartilage, CS-E from squid cartilage and DS from porcine skin were purchased from Seikagaku, Corp. (Tokyo, Japan). Hyaluronic acid (HA) from *Streptococcus equi* (15–30 kDa), CS-A from bovine trachea, Hep and HS from porcine intestinal mucosa, chondroitinase ABC (CSase ABC) (EC 4.2.2.20/21), 2-aminobenzamide (2-AB) and cyanoborohydride (NaBH_3_CN) were purchased from Sigma-Aldrich, Inc. CS tetrasaccharides were prepared by digestion of CS-D using CSase ABC, followed by gel-filtration chromatography in a Superdex^TM^ Peptide 10/300 GL column, as described previously (Wang et al., 2015). Formula A reef salt was purchased from Qingdao Goe Haida Sea Salt, Co., Ltd. (Qingdao, China). All other chemicals and reagents were of the highest quality available.

**Table 1:** Bacterial strains and plasmids.

Strains and plasmids	Description	Source
**Strains**		
*Photobacterium* sp. FC615	A CS-degrading marine bacterium (patented as CGMCC No. 16918)	
*E. coli* BL21 (DE3)	*F-, ompT, hsdSB (rB-, mB-), dcm, gal, λ (DE3), pLysS, Cm^r^*	Novagen
**Plasmids**		
pET-30a	Expression vector; kanamycin-resistant	Invitrogen
pET-30a-PB2SF	pET-30a, carrying the recombinant protein of PB2SF	

### Isolation of Marine CS-Degrading Bacteria

Coastal sediment samples were collected from Jiaozhou Bay, near Qingdao city in Shandong province, China. Artificial seawater was prepared by dissolving 100 g of formula A reef salt in 3000 ml of water. The seawater was subsequently filtered through a 0.45 μm membrane filter. Sole carbon source culture medium was prepared by dissolving 0.05% (w/v) CS-C, 0.15% (w/v) (NH_4_)_2_SO_4_, and 1.5% (w/v) agar in artificial seawater at pH 7.2. After incubation at 30°C for 72 h, colonies on the sole carbon source culture medium were randomly selected and transferred to fresh plates for further isolation. The polysaccharide-degrading abilities of the isolates were assayed via liquid culture medium without agar using CS-A, CS-C, CS-D, CS-E, DS, or HA as the sole carbon source at a final concentration of 0.5% (w/v). Bacterial growth was evaluated by measuring the absorbance at 600 nm. The concentrations of various GAGs in the culture medium were analyzed via the carbazole reaction (Bitter and Muir, 1962).

The genomic DNA of the CS-degrading bacterium was prepared using the Genome Extraction Kit (Tiangen Biotech, Co., Beijing, China). The bacterial 16S rRNA gene sequence was amplified with the bacterial universal primer pair 27F (5′-GAGTTTGATCCTGGCTCAG-3′) and 1492R (5′-AAGGA GGTGATCCAGCC-3′). Gel-recovered polymerase chain reaction (PCR) products were cloned into the pEASY-Blunt Simple Cloning Vector (TransGen) for sequencing. The sequence was analyzed against the GenBank database using the online BLAST program to search for the most similar sequences, as described in previous papers (Han et al., 2014). A phylogenetic tree was generated using the neighbor-joining method of Kumar and coworkers with MEGA version 7.0 (Kumar et al., 2016).

### Sequence Analysis of Genes and Proteins

The draft genome of *Photobacterium* sp. FC615 was sequenced at Meiji Biotech, Inc. (Shanghai, China) using Roche Applied Science 454 GsFLX, Illumina GAIIx technology. The sequence of *Photobacterium* sp. FC615 was annotated at Oak Ridge National Laboratory using their genome annotation pipeline. This included the application of a number of annotation programs, beginning with open reading frames (ORF) prediction using Prodigal, followed by manual annotation using the JGI Gene PRIMP pipeline. Automated protein function prediction was then performed using a number of databases, including the protein domains (Pfam), UniProt, TIGRFAMs, KEGG, InterPro, and COG databases; metabolic reconstruction analysis was conducted using PRIAM; tRNA prediction was carried out using tRNAscan-SE; and rRNA prediction was performed using RNAmmer as described (Han et al., 2014).

The GC content of the PB2SF ORF was calculated using BioEdit version 7.0.5.3. The molecular mass of the protein was estimated with the peptide mass tool on the ExPASy server of the Swiss Institute of Bioinformatics. Secretion signal peptides were identified using the SignalP 4.1 server. A search for similar sulfatase sequences was performed with the BLASTp algorithm online. Multiple sequence alignment and phylogenetic analysis were performed using MEGA.

### Heterologous Expression of the PB2SF Gene

To express PB2SF in *E. coli* strains, the full-length gene without signal peptide was amplified using the primers 5′-CATATGTC CCCGGCTACTCAAACAACC-3′ (forward) and 5′-CTCGAG AGTCACTGTGTCAGCCTCTTTG-3′ (reverse). Primer pairs containing restriction enzyme sites were designed according to the insert site sequences of the expression plasmid pET-30a (+) (Invitrogen). PCR was carried out using high-fidelity Prime STAR^TM^ HS DNA polymerase (Takara, Dalian, China). Gel-recovered PCR products were cloned into the expression vector pET-30a (+) following the T7 promoter, and a His_6_ tag was added at the C-terminus of the protein. The recombinant expression plasmid (pET-30a-PB2SF) was transferred to *E. coli* BL21 (DE3) cells. The integrity of the nucleotide sequence of the constructed recombinant plasmid was confirmed via DNA sequencing.

*E. coli* BL21 cells harboring the recombinant plasmid (pET-30a-PB2SF) were cultured in 100 ml of LB broth at 37°C. After the cell density reached an A_600_ of 0.8–1.0, the inducer isopropyl 1-thio-β-D-galactopyranoside was added to the broth at a final concentration of 0.05 mM to initiate the expression of the target protein. After 24 h of additional cultivation at 16°C, the cells were harvested via centrifugation at 8000 *g* for 10 min, washed twice using ice-cold buffer A [50 mM Tris-HCl, 150 mM NaCl (pH 8.0)], resuspended in buffer A, and disrupted through sonication (50 repetitions, 5 s) in a low-temperature environment. The crude enzymes were collected after centrifugation at 15,000 *g* for 30 min.

To purify PB2SF, the soluble crude enzyme was loaded onto a column packed with nickel-Sepharose^TM^ 6 Fast Flow (GE Healthcare). Then, the column was washed with buffer A containing 50 mM imidazole to remove impurities, and PB2SF was finally eluted from the Ni-NTA column with buffer A containing 200 mM imidazole. The eluate containing PB2SF was concentrated using an Amicon Ultra 0.5-ml 10K unit (Millipore) and then loaded onto a Superdex^TM^ 200 10/300 GL column and eluted with buffer A at a flow rate of 0.4 ml/min. The absorbance of the eluates was monitored at 280 nm, and the largest peak was collected. The purified protein was analyzed through SDS-PAGE in 13.2% polyacrylamide gels according to Sambrook and Russell (2006). Coomassie brilliant blue R-250 was used to stain the proteins in gels, and the protein concentration was determined via the Folin-Lowry method (Bramhall et al., 1969).

### Substrate Specificity Assay for PB2SF

To determine the substrate specificity of the enzyme, different types of unsaturated disaccharides were dissolved in deionized water to prepare stock solutions (300 pmol/μl) including the ΔC unit [Δ^4,5^HexUAβ1-3GalNAc (6S)], ΔA unit [Δ^4,5^HexUAβ1-3GalNAc (4S)], ΔD unit [Δ^4,5^HexUA(2S)β1-3GalNAc(6S)], and ΔT unit [Δ^4,5^HexUA(2S)β1-3GalNAc(4S,6S)], as well as Hep/ HS unsaturated disaccharides [Δ^4,5^HexUAα1-4GlcNAc(6S), Δ^4,5^HexUAα1-4GlcNS, Δ^4,5^HexUAα1-4GlcNS(6S), Δ^4,5^Hex UA(2S)α1-4GlcNS, and Δ^4,5^HexUA(2S)α1-4GlcNS(6S)]. Each stock solution (10 μl) was mixed with 30 μl of 150 mM Na_2_HPO_4_-NaH_2_PO_4_ buffer (pH 8.0), 40 μl of deionized water and 10 μl of appropriately diluted enzyme (2 μg), and the solutions were incubated at 30°C for 12 h. Each enzymatic reaction included a negative control in which the active enzyme was substituted with boiled inactive enzyme. Following the enzymatic reaction, the sulfatase-treated disaccharide solutions were heated in boiling water for 10 min and then cooled in ice-cold water for 5 min. Thereafter, the reaction solutions were centrifuged at 15,000 *g* for 10 min, and the supernatants were collected and labeled with 2-AB in the presence of sodium cyanoborohydride, as described by Bigge et al. (1995). Next, Free 2-AB was removed through extraction with chloroform. The labeled samples were individually analyzed via anion-exchange HPLC in a YMC-Pack PA-G column (YMC-Pack PA, Kyoto, Japan) and were eluted with a linear gradient from 16 to 460 mM NaH_2_PO_4_ over 60 min at a flow rate of 1.0 ml/min at room temperature. The eluates were monitored by measuring the absorbance using a fluorescence detector at excitation and emission wavelengths of 330 and 420 nm, respectively, as described previously (Wang et al., 2015). Disaccharides were identified and quantified based on comparison with authentic unsaturated disaccharides.

To investigate whether this enzyme acts on the saturated hexuronate-2-*O*-sulfate, a CS tetrasaccharide, Δ^4,5^HexUAβ1-3GalNAc(4S)β1-4GlcUA(2S)β1-3GalNAc(6S), was prepared as described in Section “Materials and Bacterial Strains” and was used as a substrate for the activity assay described above for disaccharides.

### Enzymatic Characterization of PB2SF

To determine the optimal temperature for enzyme activity, an aliquot of the ΔD unit (20 nmol) was incubated with 0.1 μg of PB2SF in 50 mM NaH_2_PO_4_-Na_2_HPO_4_ buffer (pH 8.0) in a total volume of 100 μl at temperatures ranging from 0 to 70°C for 10 min. After the optimum temperature was achieved, the optimal pH assay was carried out in buffers with different pH levels, including 50 mM HAc-NaAc buffer (pH 5.0–7.0), 50 mM NaH_2_PO_4_-Na_2_HPO_4_ buffer (pH 6.0–8.0), and 50 mM Tris-HCl buffer (pH 7.0–10.0) at 40°C for 10 min. The effects of metal ions or a chelating agent (5 mM) and the NaCl concentration (0–1 M) on the reaction rate of PB2SF were investigated under optimal reaction conditions (50 mM HAc-NaAc buffer pH 6.0 at 40°C). Finally, to investigate the thermostability of PB2SF, the enzyme in 50 mM HAc-NaAc buffer (pH 6.0) was preincubated at 0–60°C for 30–120 min, after which the residual activity was determined under optimum conditions. All reactions were performed in triplicate, and enzyme-treated samples were analyzed via anion-exchange HPLC in a YMC-Pack PA-G column (YMC-Pack PA, Kyoto, Japan) and eluted with a linear gradient from 16 to 460 mM NaH_2_PO_4_ over 60 min at a flow rate of 1.0 ml/min. The eluates were monitored by measuring the absorbance at 232 nm (Yamagata et al., 1968). The effects of pH and chemical reagents on the activity of PB2SF at 25°C were analyzed using the same method as described for 40°C.

After biochemical characterization of PB2SF, its enzymatic activity against ΔD unit disaccharides was determined in the optimum buffer (50 mM HAc-NaAc buffer, pH 6.0) at 40 and 25°C. One unit of enzyme was defined as the amount of enzyme required to produce 1 μmol of free sulfate per minute.

### Tertiary Structure Modeling and Key Catalytic Amino Acid Analysis of the PB2SF

The protein sequence of PB2SF was uploaded, and a three-dimensional model of PB2SF was built by the protein structure homology-modeling server SWISS-MODEL online^1^ (Biasini et al., 2014) using the structure of bacterial 2-*O*-sulfatase BT_1596 as a template (PDB code: 3b5q) (Ulmer et al., 2014). Structural figures were created and rendered using the program PyMOL (Schrödinger LLC^2^).

Mutants of PB2SF were produced by using the Fast Mutagenesis System Kit (TransGen) as described by Prabhakar et al. (2005). The primer sequences used for the mutations are presented in Table 2. The plasmids of the mutants were amplified by transferring them to DMT Chemically Competent Cells (TransGen), and clones were sequenced at Sangon Biotech, Co., Ltd. (Shanghai, China) to confirm the successful introduction of the mutations. Then, correct plasmids were transferred into *E. coli* BL21 (DE3) cells and expressed, and the enzymatic activity of the mutants was determined under optimum conditions [50 mM HAc-NaAc buffer (PH 6.0), 40°C], as described above for the wild-type enzyme PB2SF.

**Table 2:** Primer sequences for PB2SF site-directed mutagenesis.

Mutants	Primer sequence
D52A	5′-TGATCATTACCGCC**GCG**CAGCTATCTCGCC-3′
	5′-GGATGTTTTTGGGTTTGCTGGTTGTTTGAG-3′
N113A	5′-ATGGCGTGCATGGT**GCG**AACAGCCCAAAC-3′
	5′-TTTGATGCGGCAGTCGACCAGTCCAGAACG-3′
K141A	5′-CCGTCCATTTCGGC**GCG**CAACATGACTTTGG-3′
	5′-CATCATAGCCAGCTTCGCGGAATAATTGGC-3′
K141H	5′-CCGTCCATTTCGGC**CAU**CAACATGACTTTGG-3′
	5′-CATCATAGCCAGCTTCGCGGAATAATTGGC-3′
H143A	5′-ATTTCGGCAAGCAA**GCG**GACTTTGGCTCCTTG-3′
	5′-GGACGGCATCATAGCCAGCTTCGCGGAATAATTG-3′
H143K	5′-ATTTCGGCAAGCAA**AAA**GACTTTGGCTCCTTG-3′
	5′-GGACGGCATCATAGCCAGCTTCGCGGAATAATTG-3′
H205A	5′-ATTTCAACAACCCA**GCG**AATATTTGTGGTTG-3′
	5′-CAACGGCCATAATGAAAGGTTTATCGTG-3′
H205K	5′-ATTTCAACAACCCA**AAA**AATATTTGTGGTTG-3′
	5′-CAACGGCCATAATGAAAGGTTTATCGTG-3′
H310A	5′-TCTTCTTCAGTGAT**GCG**GGCGATGCCATGGG-3′
	5′-CAATCAGCGTATTGTCGGCAGCATCTGACTG-3′
H310K	5′-TCTTCTTCAGTGAT**AAA**GGCGATGCCATGGG-3′
	5′-CAATCAGCGTATTGTCGGCAGCATCTGACTG-3′

*Mutation codons in order to create the desired point mutations are underlined and indicated in bold.*

### Prediction of Δ^4,5^Hexuronate- 2-*O*-Sulfatases

The sequences of *N*-acetylgalactosamine-4-*O*-sulfatase (endoVB4SF, GenBank^TM^ Accession No. AJK90566) (Wang et al., 2015), *N*-acetylgalactosamine-4-*O*-sulfatase (BT_3349, AAO78 455), Δ^4,5^hexuronate-2-*O*-sulfatase (BT_1596, NP_810509) (Ulmer et al., 2014), Δ^4,5^hexuronate-2-*O*-sulfatase (Phep_2825, WP_015808635) (Myette et al., 2003), and *N*-acetylgalactosamine-6-*O*-sulfatase (BT_3333, NP_812245) (Ulmer et al., 2014) were downloaded from the NCBI protein sequence database.

Similarity searches of sulfatase sequences were performed with the BLASTp program using query sequences of reported GAG 2-*O*-sulfatases Phep_2825, PB2SF, and BT_1596. One hundred sequences with greater than 40% identity to each query gene were downloaded from the NCBI protein database. Among the retrieved sequences, some from different strains even species were actually the same gene. To simplify the analysis, only one of identical sequences was reserved and other “redundant identical sequences” were manually deleted. Finally, a total of 60 potential Δ^4,5^hexuronate-2-*O*-sulfatases were ultimately obtained. Then, the multiple sequence alignments were carried out using ClustalW and the phylogenetic analysis was performed using the neighbor-joining method in MEGA.

To confirm the activity of these potential 2-*O*-sulfatases, one (GenBank^TM^ Accession No. WP_083232406) of these enzymes with relatively low identity to PB2SF and the two other reported Δ^4,5^hexuronate-2-*O*-sulfatases were recombinantly expressed in the expression plasmid pET-30a (+) and characterized as described for PB2SF above.

### Signature Sequence Analysis of the Δ^4,5^Hexuronate-2-*O*-Sulfatase Family

To investigate the conserved signature regions of Δ^4,5^hexuronate-2-*O*-sulfatases, the sequences of bacterial CS/DS sulfatases, including three characterized 2-*O*-sulfatases (PB2SF from *Photobacterium* sp. FC615, BT_1596 from *Bacteroides thetaiotaomicron* VPI-5482 and Phep_2825 from *Pedobacter heparinus* DSM 2366), 60 potential 2-*O*-sulfatase genes predicted as described above and 234 sequences of family S1_9 downloaded from database SulfAtlas, were aligned using BioEdit. Then, highly conserved signature regions were selected, and logo signature sequences were built by WebLogo^3^ (Crooks et al., 2004).

## Results

### Isolation of a CS-Degrading Marine Bacterium

**FIGURE 1:**
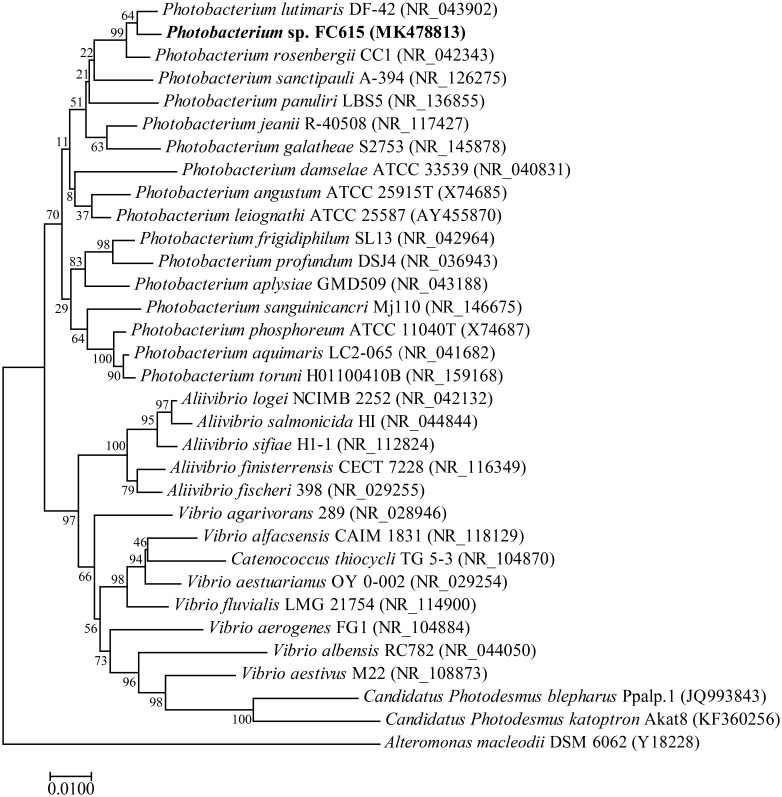
Phylogenetic position of *Photobacterium* sp. FC615 based on the 16S rRNA gene sequences. The phylogenetic analysis was performed using the neighbor-joining method in MEGA, and associated taxa clustered together in the bootstrap test of 1000 replicates.

A novel bacterium strain was isolated from marine sediment using shark cartilage CS-C as the sole carbon source. BLASTn analysis of the 16S rRNA gene (GenBank^TM^ Accession No. MK478813) showed that this bacterium belonged to the *Photobacterium* genus (Figure 1). The bacterium grew vigorously in simple medium with CS-A, CS-C, CS-D, CS-E, DS, and HA. Carbazole reaction analysis showed that the concentrations of various GAGs in the medium were dramatically decreased after culturing for 24 h (data not shown), suggesting that *Photobacterium* sp. FC615 can efficiently utilize various GAGs as a sole carbon source and contains GAG-degrading enzymes.

### Features of PB2SF Gene and Protein Sequence

To identify novel GAG-degrading enzymes, the genome of this isolated *Photobacterium* sp. FC615 was sequenced. The draft genome was ∼6.3 Mb in size and possessed at least 6400 ORFs, among which three chondroitinases and 27 sulfatase genes were predicted. One putative sulfatase gene *pb2sf* (GenBank^TM^ Accession No. MH321063), which was coded by ORF 2351, was 1533 bp in length, with 52.8% GC content (G + C %), and encoded a protein (PB2SF) with a molecular weight of ∼56.8 kDa. The theoretical isoelectric point (pI) of this protein was 5.63. SignalP 4.1 server analysis revealed the presence of an N-terminal signal peptide containing 33 amino acid residues.

A BLASTp search revealed that PB2SF shared relatively low sequence identity with most identified GAG sulfatases except for the 2-*O*-sulfatase BT_1596 from *Bacteroides thetaiotaomicron.* It shared the highest sequence identity (46%, query cover 90%) with BT_1596 (GenBank^TM^ Accession No. NP_810509) (Ulmer et al., 2014). In addition, it shared 32% sequence identity (query cover 86%) with the 2-*O*-sulfatase Phep_2825 from *Pedobacter heparinus* DSM 2366 (Myette et al., 2003) and only 25% sequence identity (query cover 72%) with human iduronate-2-*O*-sulfatase (S1_7; AAA92014) (Demydchuk et al., 2017).

### Molecular Cloning and Recombinant Expression of PB2SF

**FIGURE 2:**
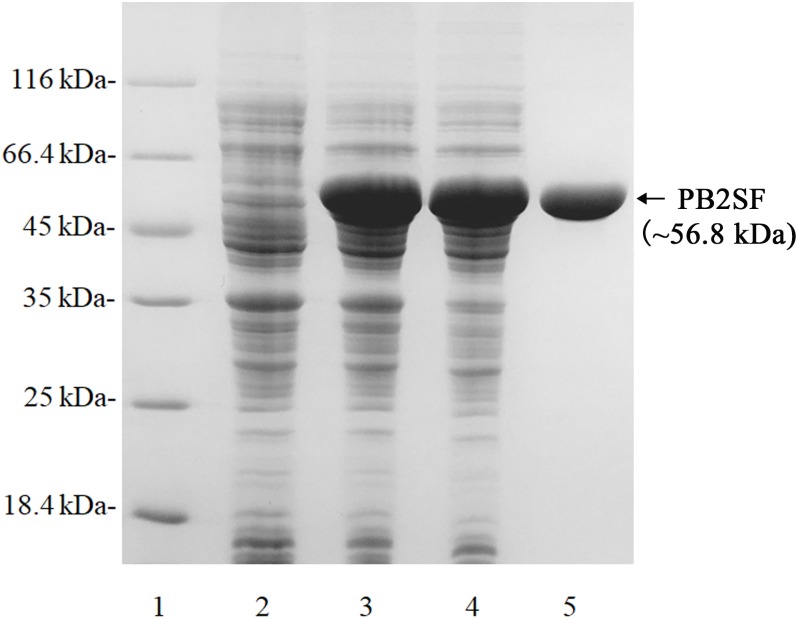
SDS-PAGE of the recombinant PB2SF from *E. coli*. PB2SF was purified through Ni^2+^-chelation chromatography and gel-filtration chromatography. Enzyme purity following each fractionation step was assessed by SDS-PAGE using 13.2% polyacrylamide gels, followed by staining with Coomassie brilliant blue. Lane 1, unstained protein molecular weight marker SM 0431 (Thermo); Lane 2, uninduced cell lysate; Lane 3, induced cell lysate; Lane 4, supernatant of the induced cell lysate; Lane 5, purified recombinant PB2SF.

The recombinant protein PB2SF was expressed in *E. coli* BL21 (DE3) cells and analyzed using SDS-PAGE (Sambrook and Russell, 2006). The results showed that BL21 (DE3) cells harboring the pET-30a-PB2SF plasmid yielded soluble protein (∼2 g/L). The protein was purified via nickel-nitrilotriacetic acid affinity chromatography and gel-filtration chromatography. SDS-PAGE showed that the purity of the PB2SF protein was greater than 98%, and the initial protein concentration was ∼5 mg/ml (Figure 2).

### Enzymatic Activity of PB2SF Toward Oligosaccharides

**FIGURE 3:**
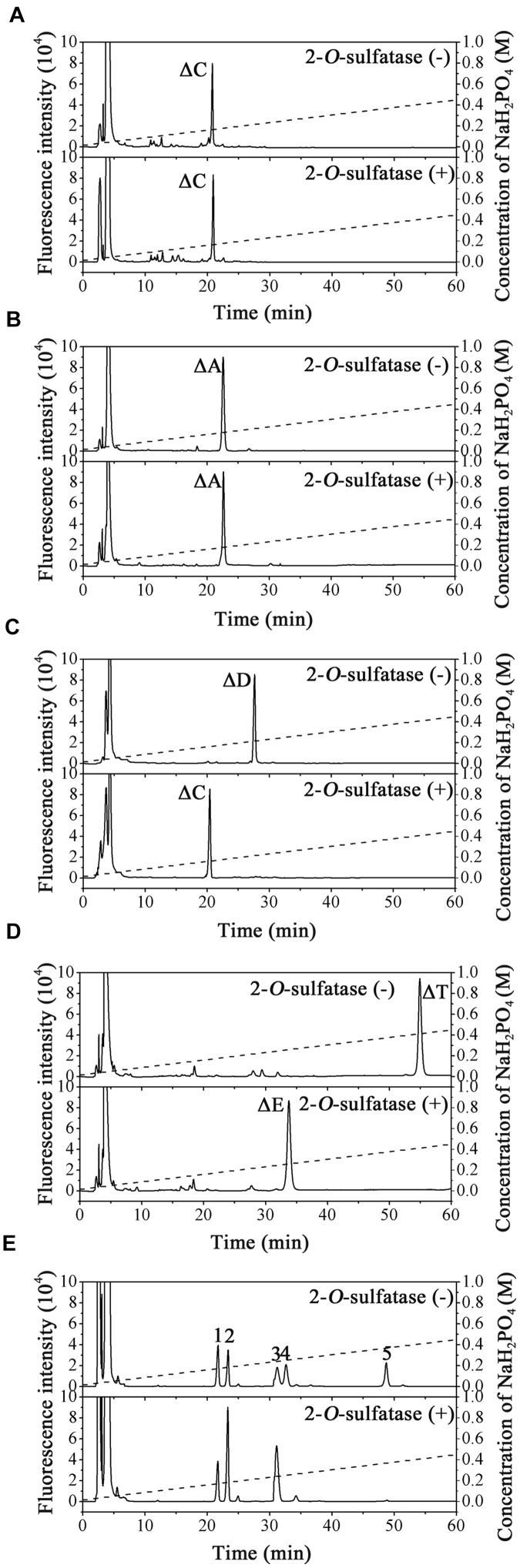
Analysis of the final products of unsaturated GAG disaccharides treated with PB2SF. Unsaturated CS/DS disaccharides ΔC **(A)**, ΔA **(B)**, ΔD **(C)**, and ΔT **(D)** and unsaturated Hep/HS disaccharides **(E)** were incubated without (top) or with (bottom) PB2SF, labeled with 2-AB and then analyzed via anion-exchange HPLC, as described in Section “Materials and Methods.” In **(A**–**D)**, the elution positions of the standard disaccharides are indicated: ΔO (Δ^4,5^HexUAβ1-3GalNAc), ΔC [Δ^4,5^HexUAβ1-3GalNAc(6S)], ΔA [Δ^4,5^HexUAβ1-3GalNAc(4S)], ΔD [Δ^4,5^HexUA(2S)β1-3GalNAc(6S)], ΔE [Δ^4,5^HexUAβ1-3GalNAc(4S,6S)], and ΔT [Δ^4,5^HexUA(2S)β1-3GalNAc(4S,6S)]. In **(E)**, the elution positions of the following standard disaccharide components are indicated by numbers, (1) Δ^4,5^HexUAα1-4GlcNAc(6S); (2) Δ^4,5^HexUAα1-4GlcNS; (3) Δ^4,5^HexUAα1-4GlcNS(6S); (4) Δ^4,5^HexUA(2S)α1-4GlcNS; and (5) Δ^4,5^HexUA(2S)α1-4GlcNS(6S).

The substrate specificity of PB2SF was characterized using unsaturated CS/DS disaccharides with different sulfation patterns. The results showed that the ΔC and ΔA units were not altered (Figure 3A,B), but the ΔD unit (Figure 3C, top) and ΔT unit (Figure 3D, top) were completely transformed to a ΔC unit (Figure 3C, bottom) and ΔE unit (Figure 3D, bottom), respectively. Likewise, the 2-*O*-sulfate groups on the unsaturated hexuronic acid of Hep/HS disaccharides [Δ^4,5^HexUA(2S)α1- 4GlcNS, and Δ^4,5^HexUA(2S)α1-4GlcNS(6S)] were also selectively removed by this enzyme (Figure 3E, bottom). These results demonstrate that the enzyme is a hexuronate- 2-*O*-sulfatase.

To determine enzyme activity toward the inner 2-*O*-sulfate esters of oligosaccharides, the CS-D-derived tetrasaccharide Δ^4,5^HexUAβ1-3GalNAc(4S)β1-4GlcUA(2S)β1-3GalNAc(6S) was used as a substrate for treatment with PB2SF. PB2SF did not affect the sulfation pattern of this tetrasaccharide (Supplementary Figure S1), indicating that this enzyme is a strict exo-sulfatase that specifically catalyzes the hydrolysis of 2-*O*-sulfate on the Δ^4,5^hexuronate residues located at the non-reducing end of GAG chains and is a Δ^4,5^hexuronate-2-*O*-sulfatase.

### Biochemical Properties of the PB2SF

**FIGURE 4:**
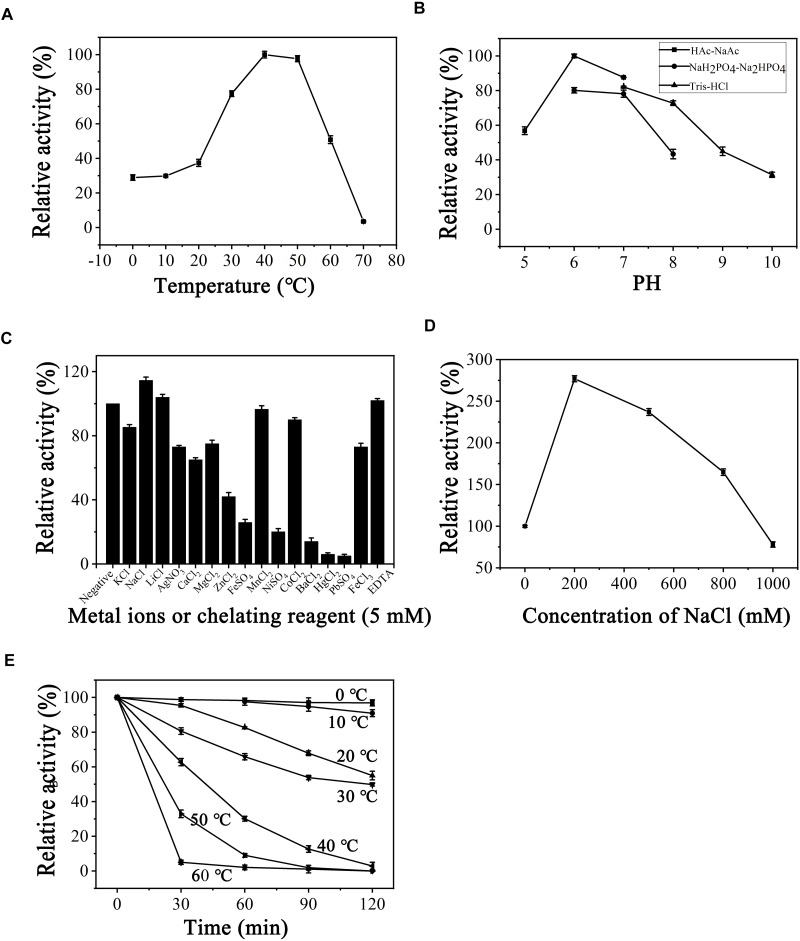
Biochemical conditions for recombinant PB2SF. **(A)** Effect of temperature. The enzyme activity of PB2SF was measured using the ΔD disaccharide as a substrate in 50 mM NaH_2_PO_4_-Na_2_HPO_4_ buffer (pH 8.0), at different temperatures for 10 min. The data are shown as percentages of the activity obtained at 40°C (100%). **(B)** Effect of pH. The activity of PB2SF against ΔD was measured in buffers with pH values from 5 to 10 at 40°C for 10 min. The data are shown as percentages of the activity obtained in the HAc-NaAc buffer at pH 6.0 (100%). **(C)** Effects of metal ions. The activities of PB2SF against ΔD were measured in the HAc-NaAc buffer (pH 6.0) containing 5 mM of various metal ions at 40°C for 10 min. Data are shown as a percentage of the activity obtained in the buffer without the tested metal ions. **(D)** Effects of NaCl concentrations. The activity of PB2SF against ΔD was measured in HAc-NaAc buffer (pH 6.0) containing 0–1 M NaCl at 40°C for 10 min. Data are shown as a percentage of the activity obtained in the buffer without NaCl. **(E)** Thermostability of PB2SF. The enzyme in 50 mM HAc-NaAc buffer (pH 6.0) was preincubated for 0–120 min at temperatures from 0 to 60°C, and the residual activity against ΔD was estimated at 40°C. Data are shown as the activity relative to that of untreated PB2SF. Error bars represent the means of triplicate analyses ± SD.

After determining substrate specificity, the optimal catalytic reaction conditions for PB2SF were further defined. Using the ΔD unit as a substrate, enzymatic reactions were carried out under different conditions, varying single factors, including temperature, pH, and the chemical components, as described in Section “Materials and Methods.” PB2SF exhibited the highest reaction rate at 40°C, and the rate did not vary greatly from 40 to 50°C (Figure 4A). The effect of the pH on enzymatic activity was investigated at the optimum temperature (40°C). The optimum pH was 6.0 in 50 mM HAc-NaAc buffer (Figure 4B). Notably, the PB2SF maintained relatively high activity in acidic buffer, especially in HAc-NaAc buffer. To determine the influence of metal ions on enzymatic activity, various metal ions and EDTA were individually added to the reaction buffer at a final concentration of 5 mM. Na^+^ and Li^+^ promoted enzymatic activity, but Ni^2+^, Ba^2+^, Hg^2+^, and Pb^2+^ strongly inhibited activity. In addition, EDTA, a divalent metal ion-chelating agent, did not inhibit sulfatase activity (Figure 4C). Considering that the enzyme was isolated from a marine bacterium and that its activity was found to be stimulated by monovalent metal ions, such as Na^+^ or Li^+^, it may be a halophilic enzyme. Thus, we further investigated the effect of the salt concentration on enzymatic activity by increasing the concentration of NaCl from 0 to 1 M. The activity was increased by 2.7-fold when the NaCl concentration was increased from 0 to 200 mM, at which point the activity gradually decreased as the salt concentration was increased. However, the relative sulfatase activity was still much higher than that in the buffer in the absence of NaCl until the NaCl concentration reached 800 mM (Figure 4D). Finally, the thermostability of this enzyme was investigated under optimum conditions. The results showed that PB2SF was very stable at 0–10°C (Figure 4E) and it maintained >90% of its activity after pre-incubated at 4°C for 12 h (data not shown). The PB2SF protein was also relatively stable at 20–30°C and the half-life at 30°C was approximately 120 min. In contrast, the stability of the enzyme quickly decreased when the temperature reaches to ≥40°C. Based on the results (Figure 4A,E), the enzyme is much more stable and has relatively high activity at 20–30°C, which should be the preferred conditions for the practical applications of this enzyme. Thus, the effects of pH and chemical reagents on the activity of PB2SF were investigated at 25°C. The results showed that the effects of these factors on the enzyme activity were similar to those obtained at 40°C (Supplementary Figure S2). The specific activity of PB2SF, which was determined in the optimum buffer (50 mM HAc-NaAc buffer, pH 6.0) was 5.3 and 2.2 units/mg of protein at 40 and 25°C, respectively (Table 3).

**Table 3:** Activity analysis of PB2SF.

	Total protein (mg)	Total activity at 40°C (milliunits)	Specific activity at 40°C (milliunits/mg)	Total activity at 25°C (milliunits)	Specific activity at 25°C (milliunits/mg)
Crude protein	45 ± 2.8	238500	5300 ± 169	99180	2204 ± 104
Purified protein	18 ± 1.9	173574	9643 ± 180	78138	4341 ± 132

*Presented results are the means ± SD for at least two experiments.*

### Site-Directed Mutagenesis of the Catalytic Site

**FIGURE 5:**
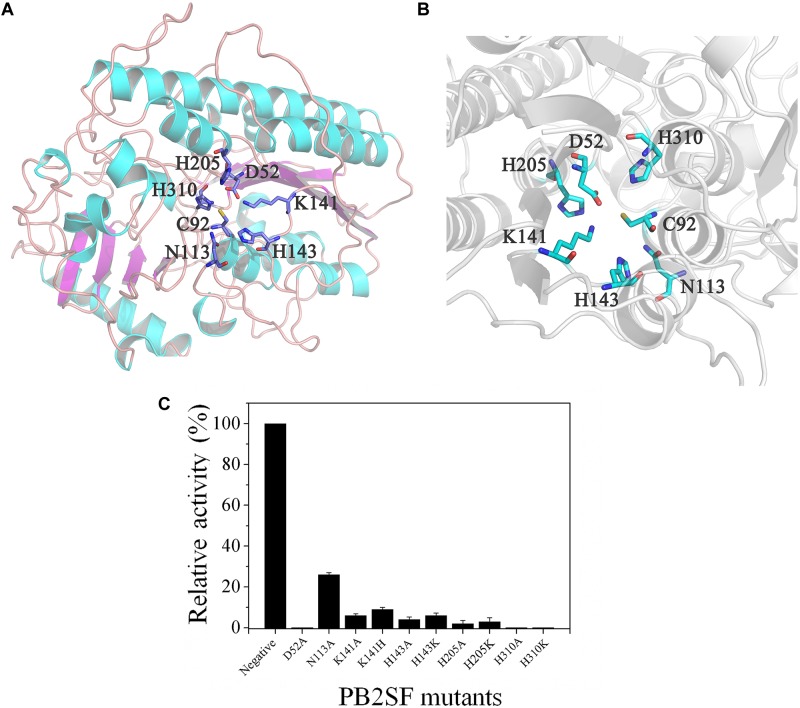
Structural modeling and catalytic residue analysis of PB2SF. **(A)** Stereo view of the entire three-dimensional model. Amino acids around the active site are shown in stick mode. Notably, the Cys92 residue was not transformed to FGly in structural modeling, as it was in the active form after post-translational modification. **(B)** More detailed stereo view of the active site region. Figures were created with the program PyMOL. **(C)** Enzymatic activity analysis of PB2SF mutants. The activities of PB2SF mutants against ΔD disaccharides were measured in HAc-NaAc buffer (pH 6.0) at 40°C for 10 min. Data are shown as a percentage of the activity of wild-type PB2SF under optimal reaction conditions.

To analyze the key amino acids involved in the catalytic mechanism of PB2SF, a structural model of the sulfatase (Figure 5A,B) was constructed via a protein homology-modeling method using the reported structure of the bacterial 2-*O*-sulfatase BT_1596 as a template (PDB code: 3b5q), as described in Section “Materials and Methods.” According to the structural model, six potential catalytic amino acids (Asp52, Asn113, Lys141, His143, His205, and His310) around the Cys92 residue (the precursor of FGly residue) were selected and individually mutated to Ala or similar residues to probe their roles in the catalytic mechanism. Analysis of the mutants’ enzymatic activities revealed that only the D52A, H310A, and H310K mutants were totally inactivated, indicating a key role of Asp52 and His310 in the catalytic mechanism (Figure 5C). Additionally, the mutation of His205 to Ala or Lys (H205A or H205K) caused a dramatic decrease in enzymatic activity to less than 5% of the wild-type activity. Notably, although His and Lys are both basic amino acids, the severe reduction in activity observed upon replacing His205 with Lys suggests that the imidazole group of His205, an important nucleophilic group, is essential to the catalytic mechanism. The Lys141 and His143 mutants (K141H, K141A, H143A, and H143K) also exhibited low activity, indicating that both of these sites are also involved in the catalytic mechanism. Taken together, the identification of these key residues provides a basis for future analysis of the catalytic mechanism of bacterial GAG sulfatases.

### Prediction of GAG-Specific Sulfatases via the Bioinformatic Method

**FIGURE 6:**
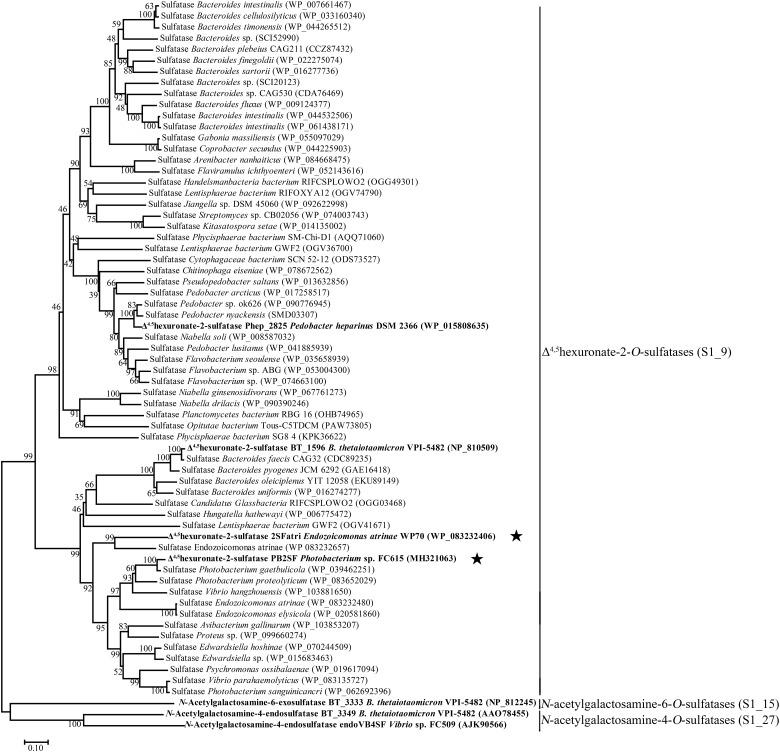
Phylogenetic analysis of the characterized bacterial CS/DS sulfatases and 60 potential 2-*O*-sulfatases. The neighbor-joining tree was constructed with MEGA. The numbers on the branches indicate the bootstrap confidence values from 1000 replicates. The bar is equal to the distance corresponding to 1 amino acid substitution per 10 amino acid residues. Sulfatases that have been previously reported are indicated in bold. PB2SF and 2SFatri elucidated in this study are labeled with five-pointed stars. Based on the classification of the database SulfAtlas, Δ^4,5^hexuronate-2-*O*-sulfatases belong to the family *S1_9*; *N*-acetylgalactosamine-4-*O*-sulfatases and *N*-acetylgalactosamine-6-*O*-sulfatases belong to the family *S1_27* (EC 3.1.6.12) and *S1_15* (EC 3.1.6.4), respectively, which are included as outgroup.

To analyze the phylogenetic relationships between different bacterial CS/DS sulfatases, the sequences of the identified bacterial 4-*O*-sulfatases, 6-*O*-sulfatases and 2-*O*-sulfatases, including PB2SF, were aligned and analyzed. The phylogenetic tree showed that sulfatases with similar specificities clustered to form a single branch, although they descended from separate ancestral genes. This information is important for the prediction of novel GAG sulfatases with specific activities based on gene sequences. Based on this finding, additional potential Δ^4,5^hexuronate-2-*O*-sulfatases were screened from the GenBank database with the BLASTp program using three query sequences, including PB2SF and the two already reported BT_1596 and Phep_2825 sequences. For each query gene, 100 homogenous sequences were extracted from the database including some identical sequences. After eliminating the redundant identical sequences, 60 candidate genes were obtained. Among the sixty genes, 39 sequences were homologous to Phep_2825, 18 sequences were homologous to PB2SF, 14 sequences were homologous to BT_1596 and 11 sequences were homogenous to both the latter query sequences. Phylogenetic analysis showed that these 60 putative sulfatases clustered with the branches of the 2-*O*-sulfatase family (Figure 6), which further indicated that they were potential Δ^4,5^hexuronate-2-*O*-sulfatases.

**FIGURE 7:**
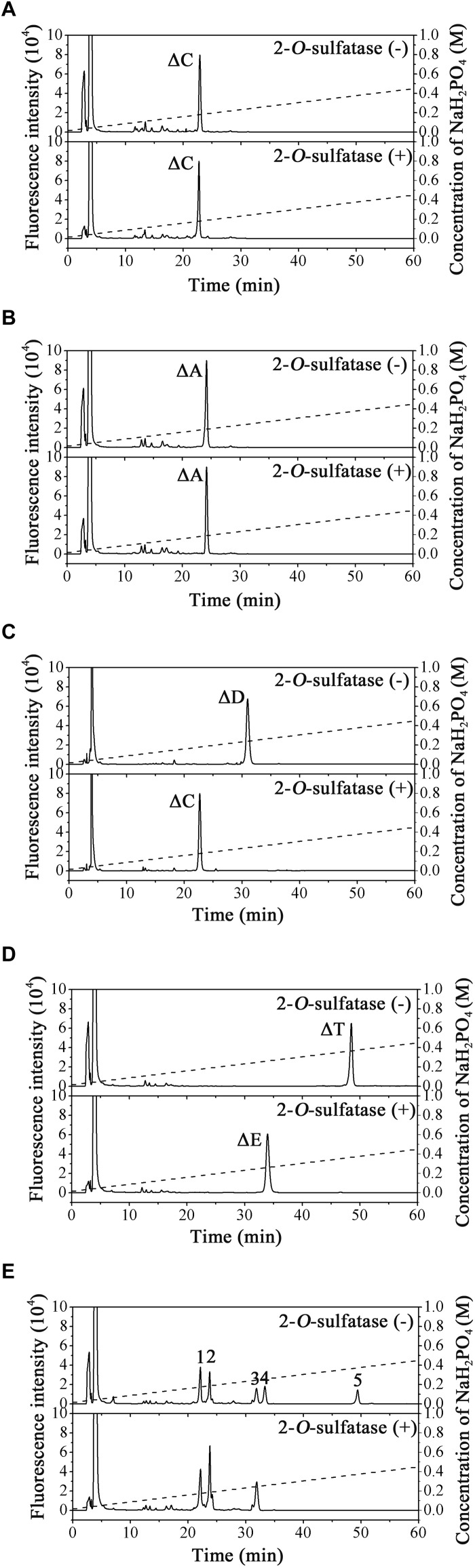
Final product analysis of unsaturated GAG disaccharides treated with 2SFatri. Unsaturated CS/DS disaccharides ΔC **(A)**, ΔA **(B)**, ΔD **(C)**, and ΔT **(D)** and unsaturated Hep/HS disaccharides **(E)** were incubated without (top) or with (bottom) 2SFatri and analyzed via anion-exchange HPLC, as described for PB2SF.

To confirm our predictions, we cloned and expressed one of these 2-*O*-sulfatase candidate genes. Notably, the putative protein from *Endozoicomonas atrinae* WP70 (GenBank^TM^ Accession No. WP_083232406), which was annotated as a DUF4976 domain-containing protein in the NCBI protein database, shared 49 and 40% sequence identity with PB2SF and BT_1596, respectively. This putative gene, designated 2SFatri, was cloned into the pET-30a (+) vector, and the recombinant protein was expressed in *E. coli* BL21 (DE3) cells. SDS-PAGE analysis showed that BL21 (DE3) cells harboring the pET-30a-2SFatri plasmid yielded soluble recombinant protein (∼1.8 g/L) (Supplementary Figure S3). The substrate specificity of 2SFatri was assessed by using various unsaturated GAG disaccharides with different sulfation patterns, as described for PB2SF above. Our experiments clearly showed that, similarly to PB2SF, 2SFatri specifically removed 2-*O*-sulfate groups from the Δ^4,5^hexuronate residue (Figure 7), which strongly supports our prediction.

### Signature Sequences of Δ^4,5^Hexuronate-2-*O*-Sulfatases

FGly-dependent sulfatases can be preliminary identified using the signature motifs PF00884 (sulfatase) or PS00523 and PS00149, proposed at PROSITE^4^. Recently, Barbeyron et al. (2016a) identified three additional conserved FGly sulfatase signatures. However, there has been no specific report about the signature sequences of GAG sulfatases.

**FIGURE 8:**
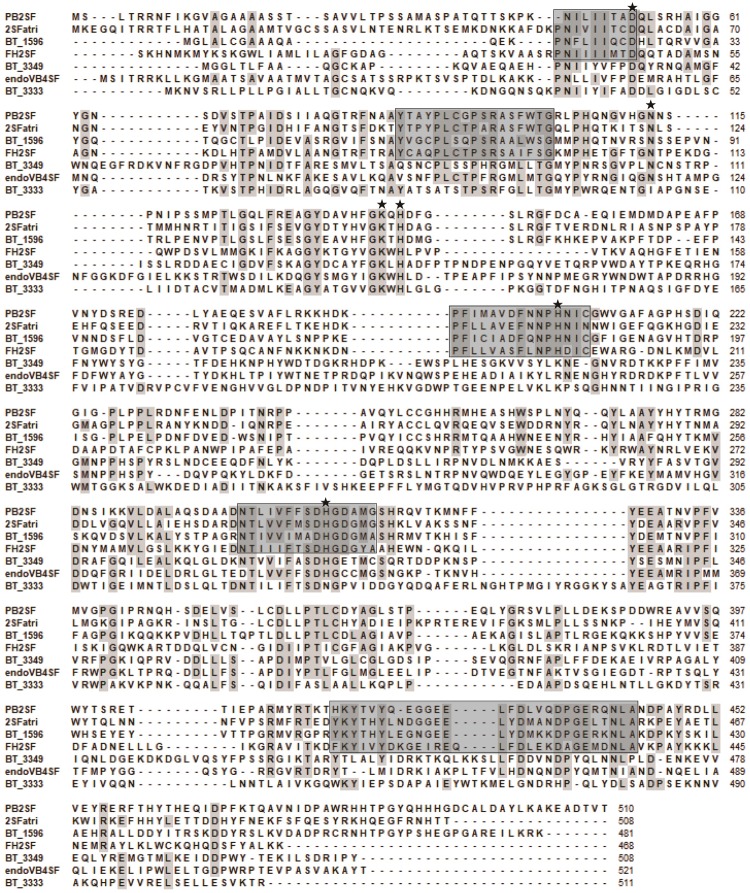
Protein sequence alignment of the characterized bacterial CS/DS sulfatases. Amino acids with partial identity are shaded in gray. Highly conservative amino acid residues of Δ^4,5^hexuronate-2-*O*-sulfatase family are indicated with black frames. Sulfatases are abbreviated as follows: BT_3349 from *Bacteroides thetaiotaomicron* VPI-5482 (GenBank^TM^ Accession No. AAO78455), endoVB4SF from *Vibrio* sp. FC509 (AJK90566), BT_3333 from *Bacteroides thetaiotaomicron* VPI-5482 (NP_812245), BT_1596 from *Bacteroides thetaiotaomicron* VPI-5482 (NP_810509), Phep_2825 from *Pedobacter heparinus* DSM 2366 (WP_015808635). The mutated amino acids (Asp52, Asn113, Lys141, His143, His205, and His310) indicated in Figure 5A,B were marked by asterisk.

**FIGURE 9:**
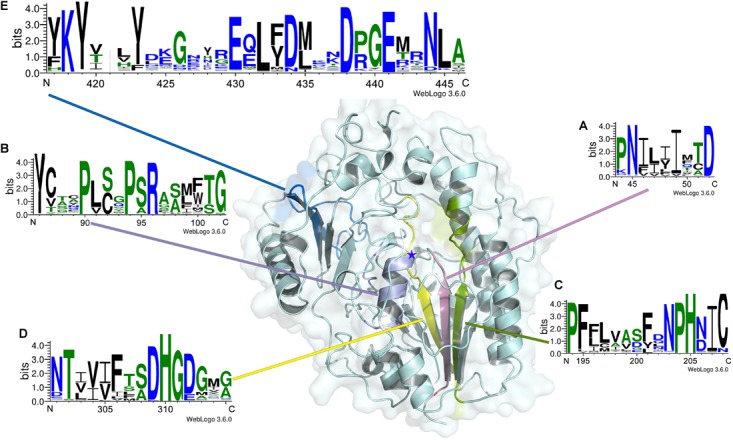
Signature sequence logos of Δ^4,5^hexuronate-2-*O*-sulfatases. Signature sequence logos of Δ^4,5^hexuronate-2-*O*-sulfatases were created with the WebLogo online server (http://weblogo.threeplusone.com/). The numbers below the logo sequences indicate, at the first position, the corresponding positions of the PB2SF protein sequence. The positions of signature sequences **(A–E)** in the structural model of PB2SF are indicated in pink, purple, green, yellow, and blue, respectively. The five-pointed star indicates the position of the key residue Cys92.

In this study, to investigate the signatures of bacterial CS/DS sulfatases, the protein sequences of CS/DS sulfatases identified from bacteria were aligned and analyzed. We found that although all the sulfatases possessed Cys/Ser-X-Pro-X-Arg as a signature sequence at the active site, the conserved sequences near the active site were distinct in each type of CS/DS sulfatase, and sulfatases with specific degradation properties possessed unique signature sequences (Figure 8). To further identify the signature sequences of Δ^4,5^hexuronate-2-*O*-sulfatases, we analyzed all identified bacterial 2-*O*-sulfatases including PB2SF, the 60 potential 2-*O*-sulfatases reported in this paper and sequences of family S1_9 predicted in SulfAtlas database. We found a series of highly conserved sequences specific to Δ^4,5^hexuronate-2-*O*-sulfatases. In the N-terminal domain, there are two conserved regions: ${\text{P}}_{44}^{70\%}{\text{-N}}_{45}^{99\%}{\text{-I}}_{46}^{74\%}{\text{-L}}_{47}^{57\%}{\text{-X}}_{48}{\text{-I}}_{49}^{95\%}{\text{-X}}_{50}{\text{-T}}_{51}^{63\%}{\text{-D}}_{52}^{100\%}$ and ${\text{Y}}_{86}^{100\%}-{\text{C}}_{87}^{64\%}-{\text{X}}_{88}-{\text{X}}_{89}-{\text{P}}_{90}^{100\%}-{\text{L}}_{91}^{82\%}-C/S_{92}-{\text{X}}_{93}-{\text{P}}_{94}^{100\%}$ $-{\text{S}}_{95}^{86\%}-{\text{R}}_{96}^{100\%}-{\text{X}}_{97}-{\text{S}}_{98}^{50\%}-{\text{X}}_{99}-{\text{X}}_{100}-{\text{T}}_{101}^{76\%}-{\text{G}}_{102}^{97\%}$ (the superscripts and subscripts denote the percentage of conservation in the alignment and the corresponding amino acid, respectively, at the first position of PB2SF; the catalytic amino acid is indicated in bold; Figure 9A,B). In the middle region of the 2-*O*-sulfatase sequences, there are also two highly homologous regions: ${\text{P}}_{194}^{100\%}$ - ${\text{F}}_{195}^{95\%}{\text{-X}}_{196}-{\text{L}}_{197}^{68\%}-{\text{V}}_{198}^{52\%}-{\text{A}}_{199}^{54\%}-{\text{X}}_{200}-{\text{F}}_{201}^{73\%}-{\text{X}}_{202}{\text{-N}}_{203}^{97\%}{\text{-P}}_{204}^{100\%}$ ${\text{-H}}_{205}^{100\%}-{\text{N}}_{206}^{68\%}-{\text{I}}_{207}^{95\%}-{\text{C}}_{208}^{91\%}$ (Figure 9C) and ${\text{N}}_{301}^{78\%}-{\text{T}}_{302}^{98\%}-{\text{X}}_{303}$ $-{\text{V}}_{304}^{65\%}-{\text{I}}_{305}^{54\%}-{\text{F}}_{306}^{82\%}-{\text{X}}_{307}{\text{-S}}_{308}^{70\%}{\text{-D}}_{309}^{99\%}{\text{-H}}_{310}^{100\%}{\text{-G}}_{311}^{100\%}{\text{-D}}_{312}^{86\%}$ ${\text{-G}}_{313}^{75\%}-{\text{X}}_{314}-{\text{G}}_{315}^{58\%}$ (Figure 9D). Notably, although these four signature motifs are decentralized in the primary structure, they surround the catalytic key residue and are tightly concentrated at the catalytic center in the tertiary structure. In contrast, a long conserved sequence, ${\text{Y}}_{417}^{67\%}-{\text{K}}_{418}^{100\%}-{\text{Y}}_{419}^{100\%}-{\text{X}}_{420}-{\left(\text{X},0\right)}_{421}{\text{-X}}_{422}$ ${\text{-Y}}_{423}^{86\%}-{\text{X}}_{424}-{\text{X}}_{425}{\text{-G}}_{426}^{84\%}-{\left(\text{X},0\right)}_{427}\text{-}{\left(\text{X},0\right)}_{428}{\text{-X}}_{429}{\text{-E}}_{430}^{100\%}$ $-{\text{Q}}_{431}^{48\%}-{\text{L}}_{432}^{99\%}-{\text{F}}_{433}^{47\%}-{\text{D}}_{434}^{87\%}-{\text{M}}_{435}^{50\%}-{\text{X}}_{436}-{\text{X}}_{437}-{\text{D}}_{438}^{100\%}-{\text{P}}_{439}^{52\%}$ ${\text{G}}_{440}^{92\%}-{\text{E}}_{444}^{100\%}-{\text{X}}_{442}-{\text{X}}_{443}-{\text{N}}_{444}^{92\%}-{\text{L}}_{445}^{95\%}-{\text{A}}_{446}^{80\%}$, in the C-terminal domain (Figure 9E), located outside of the catalytic cavity, may be involved in the binding site of substrates. Overall, all of the signature sequences together contribute to the formation of the 2-*O*-sulfatase catalytic center, which indicates that these conserved regions may influence the substrate selectivity of GAG sulfatases. Therefore, we propose that these conserved regions play essential roles during catalysis and have therefore been highly conserved during biological evolution. These homologous sequences cluster onto a single branch in the phylogenetic tree.

## Discussion

Bacterial GAG sulfatases are essential enzymes for the biodegradation of sulfated GAGs and are potential tools for the structural and functional analysis of complex GAGs. However, only a few GAG sulfatases have been identified from soil and gut bacteria (Yamagata et al., 1968; Myette et al., 2009; Ulmer et al., 2014). Most sulfatases can be preliminary identified using common substrates such as 4-methylumbelliferyl sulfate (4MUS) or *p*-nitrophenyl sulfate (pNPS) (Nicholls and Roy, 1971), but further identification of GAG-specific sulfatases requires the use of GAG oligo-/polysaccharides with unique structural features as substrates. The structural complexity of substrates and the resulting products presents a large technical challenge to the study of GAG sulfatases. To identify novel enzymatic tools for studying GAG polysaccharides and enrich the database of GAG sulfatases, we searched for GAG sulfatases in marine bacteria and identified a series of novel GAG-specific sulfatases, including the first 4-*O*-endosulfatase from a marine bacterium, *Vibrio* sp. FC509 (Wang et al., 2015). The Δ^4,5^hexuronate-2-*O*-sulfatase PB2SF reported in this paper was identified from the strain *Photobacterium* sp. FC615, which was freshly isolated from coastal sediment using CS as the sole carbon source. It specifically hydrolyzes the 2-*O*-sulfate groups on the unsaturated hexuronate residues at the non-reducing end of CS/DS or Hep/HS oligosaccharides produced by GAG lyases and is similar to Phep_2825 (Myette et al., 2003). However, unlike Phep_2825 whose relative activity quickly decreased as the temperature reached 40°C, PB2SF exhibited the maximal rate at 40°C. Furthermore, the activity of Phep_2825 is strongly inhibited by NaCl concentrations exceeding 100 mM while PB2SF exhibits halophilic characteristics, which is a common feature of many marine-derived enzymes. Interestingly, divalent metal ions such as Ca^2+^, Mg^2+^, and Zn^2+^ did not increase enzymatic activity, and the chelator EDTA did not inhibit enzymatic activity at a concentration of 5 mM. This observation has also been reported in previous studies (Lukatela et al., 1998; Kozak et al., 2015). However, structural studies of various sulfatases have shown that divalent metal ions such as Ca^2+^ or Mg^2+^ (Waldow et al., 1999; Boltes et al., 2001) play a role in the catalytic center. Thus, the reason that the activities of PB2SF and other sulfatases were not affected by these metal ions in reaction buffer needs to be investigated further.

The core motif Cys/Ser-X-Pro-X-Arg is conserved in the sulfatase family S1 in SulfAtlas database, and the FGly residue is critical for the catalytic activity of sulfatases. The crystal structures of three human GAG sulfatases, arylsulfatase B (*N*-acetylgalactosamine-4-*O*-sulfatase; S1_2) (Bond et al., 1997), GALNS (*N*-acetylgalactosamine-6-*O*-sulfatase; S1_5) (Rivera-Colon et al., 2012) and IDS (Iduronate-2-*O*-sulfatase; S1_7) (Demydchuk et al., 2017), have been solved, and several key catalytic residues, including Asp, His, Lys, and Arg, have been experimentally identified in their protein structures (Waldow et al., 1999; von Bulow et al., 2001). Recently, the cocrystal of bacterial 2-*O*-sulfatase BT_1596 (S1_9) with unsaturated CS disaccharide was reported (PDB code: 5G2T), which revealed that a series of key residues were involved in the interaction with the disaccharide substrate. However, the role of these catalytic residues remains to be verified through biochemical methods. In this study, a three-dimensional structural model of PB2SF was constructed by homology modeling, and six conserved amino acids (Asp52, Asn113, Lys141, His143, His205, and His310) as well as the key FGly residue were found to form the groove in the catalytic center. These residues are also conserved in the catalytic center of BT_1596, as revealed by its cocrystal with disaccharide substrate. Site-directed mutagenesis analysis showed that His310 and Asp52, similar to FGly, were indispensable for enzymatic activity, and the imidazole group of His310 was also critical. Additionally, the K141A, K141H, H143A, H143K, H205A, and H205K mutants exhibited minimal activity, suggesting that these basic amino acids also participate in the catalytic mechanism and together create a basic microenvironment to facilitate the binding of the negatively charged GAG chains to the active site. In contrast, the N113A mutant maintained 30% activity, indicating that the Asn113 plays a minor role in the catalytic process though it is closely adjacent to the substrate in the structure. Thus, we experimentally demonstrated the importance of conserved residues around FGly in the active site of this bacterial GAG sulfatase.

It is noteworthy that in the draft genome of *Photobacterium* sp. FC615, 27 sequences were predicted to be sulfatases, but no sequence was annotated to be a Δ^4,5^hexuronate-2-*O*-sulfatase based on the sequencing results. To discover novel GAG sulfatases, several putative sulfatase genes in the genome were recombinantly expressed and characterized, among which the 2-*O*-sulfatase PB2SF was identified. Bacterial GAG sulfatases cannot be specifically and properly annotated in very commonly used databases such as GenBank, because few bacterial GAG sulfatases have been identified, and there is a lack of systematic studies on this class of enzyme. Notably, an interrogation in professional sulfatase database SulfAtlas showed that the PB2SF gene was homologous to the genes in family S1_9 but not others, indicating the potential of the SulfAtlas for the annotation of sulfatases. However, we found that most Δ^4,5^hexuronate-2-*O*-sulfatases predicted in this study are not included in the family S1_9 of SulfAtlas, indicating that this database still needs to be improved through more studies on sulfatases in particular the identification of novel sulfatases. In this study, we found that GAG sulfatases with identical substrate specificity show a high level of sequence identity and cluster together in the phylogenetic tree. By analyzing the sequences of Δ^4,5^hexuronate-2-*O*-sulfatases, including PB2SF and 2SFatri as well as 59 other potential genes reported in this paper, five signature sequences were obtained, which were found to construct the catalytic center of the enzymes. These findings will aid in the proper prediction and identification of GAG sulfatases, particularly Δ^4,5^hexuronate-2-*O*-sulfatases, and provide important information for research on the substrate specificity of these enzymes.

*Photobacterium* is a common genus of marine bacteria that is often found in association with marine organisms such as fish (Ast and Dunlap, 2005); however, we report for the first time that this bacterium can utilize animal polysaccharide GAGs, indicating that *Photobacterium* may be involved in some physiological and pathological processes in marine animals. Indeed, various putative genes of GAG-degrading enzymes, such as lyases and sulfatases, have been found in the genomes of the pathogenic bacteria of marine fish, including *Vibrio* and *Photobacterium* (Rivas et al., 2013; Guardiola et al., 2014). Although researchers believe that these enzymes are important virulence factors of fish as well as humans (Lafisca et al., 2008), the biochemical characteristics and pathogenic mechanisms of these enzymes remain to be investigated. Our findings will provide a basis for these types of studies.

## Conclusion

Studies showing the importance of bacterial GAG sulfatases in the degradative metabolism and structural analysis of sulfated GAGs have led to an urgent demand for systematic biochemical and bioinformatic studies on various GAG sulfatases with different substrate specificities. The present study addresses this issue.

## Author Contributions

SW conducted most of the experiments, analyzed the results, and wrote the manuscript under the guidance of FL. JG conducted for sulfatase site-directed mutagenesis experiments. QZ and XC conducted the experiments involving sulfatase expression. FL conceived the idea for the project and wrote the manuscript. All authors read and approved the final manuscript.

## Conflict of Interest Statement

XC was employed by company Dongying Tiandong Pharmaceutical Company, Ltd. The remaining authors declare that the research was conducted in the absence of any commercial or financial relationships that could be construed as a potential conflict of interest.

## Footnotes

1 https://swissmodel.expasy.org/

2 http://www.pymol.org

3 http://weblogo.threeplusone.com/

4 https://prosite.expasy.org/
