# Supplementary figures and images for: Identification and Signature Sequences of Bacterial Δ4,5Hexuronate-2-O-Sulfatases
Source: Front Microbiol. 2019 Apr 5;10:704. doi: 10.3389/fmicb.2019.00704 (PMC6460246; doi:10.3389/fmicb.2019.00704)

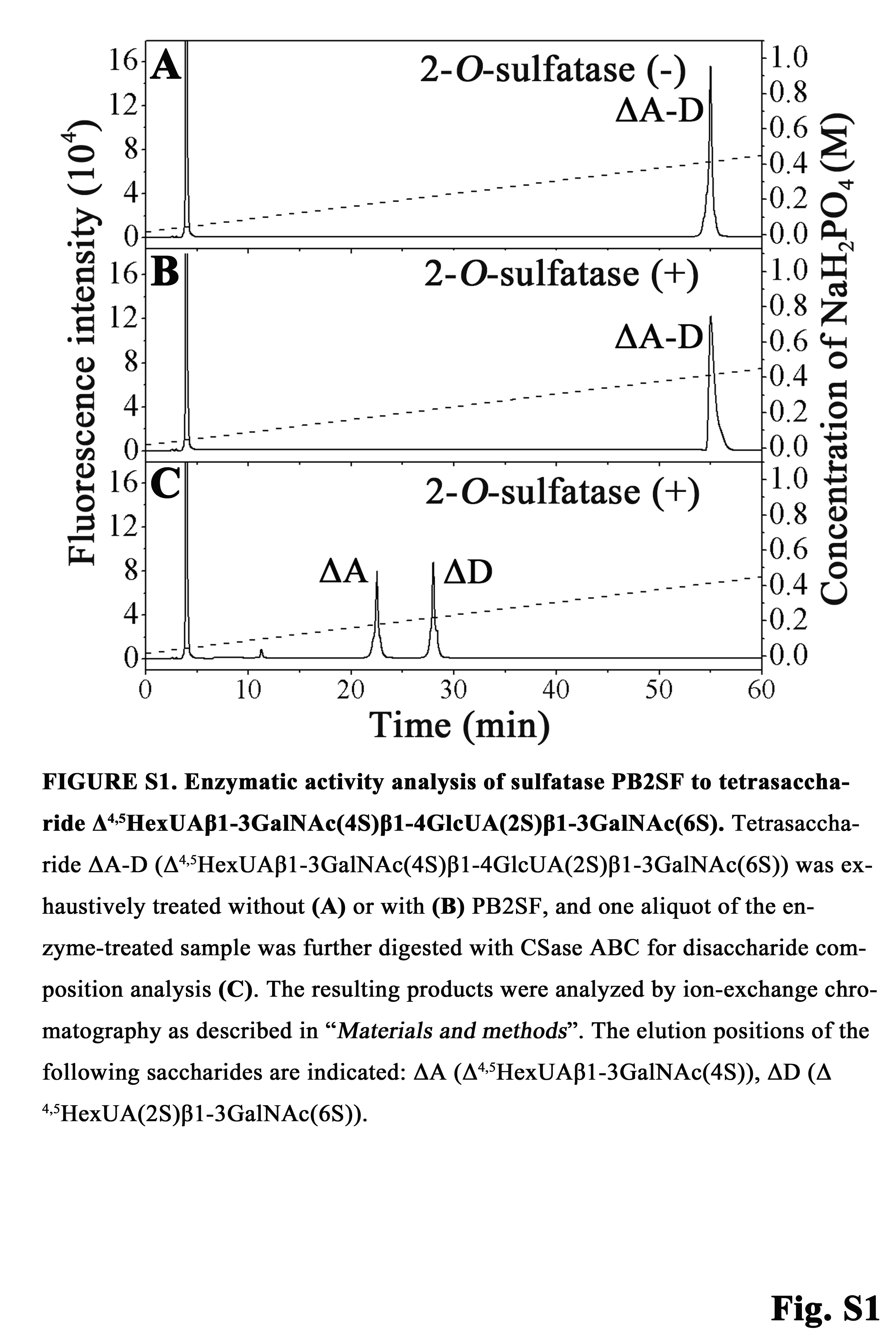

Supplement: Supplementary file 1 [file Image_1.TIF]

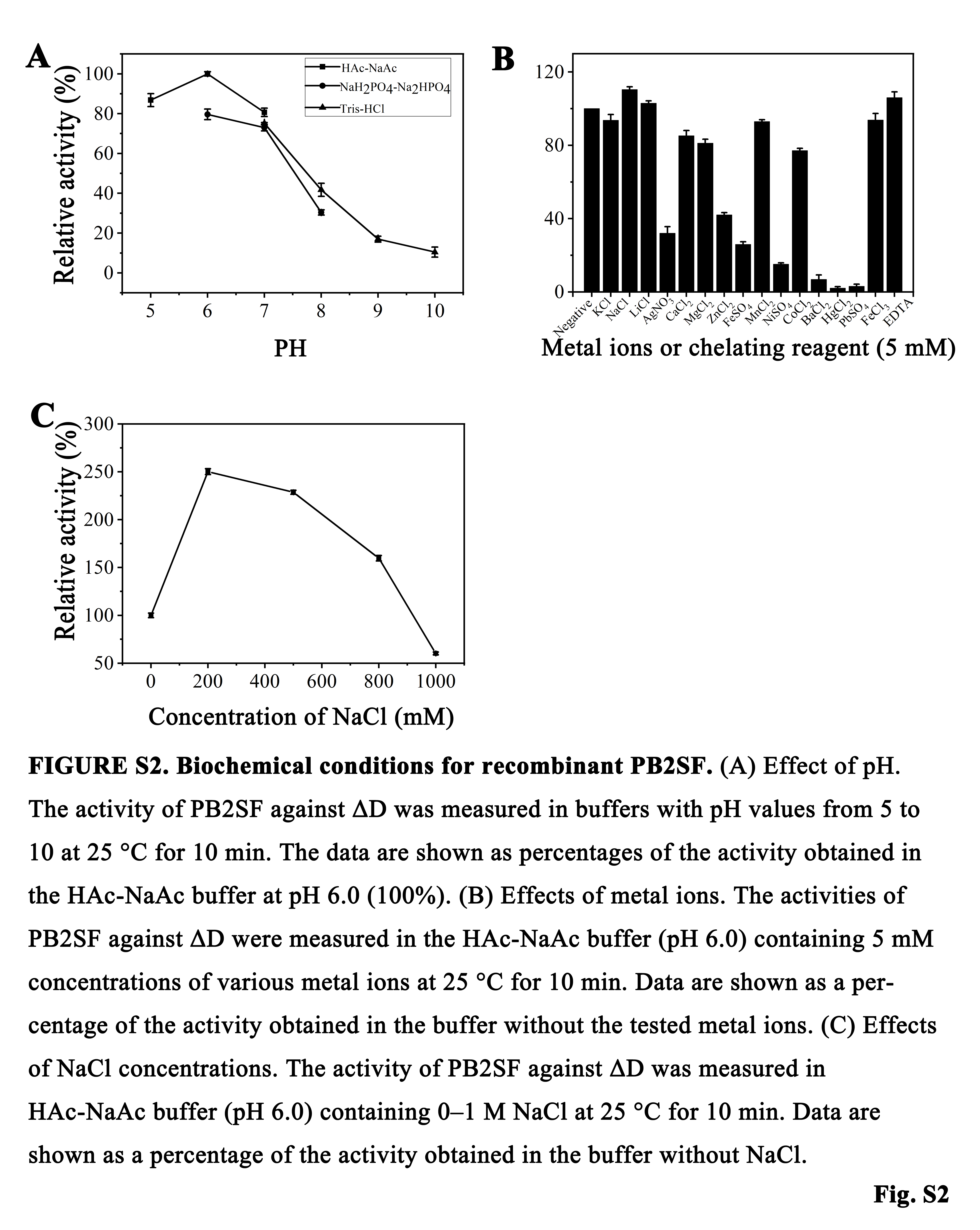

Supplement: Supplementary file 2 [file Image_2.TIF]

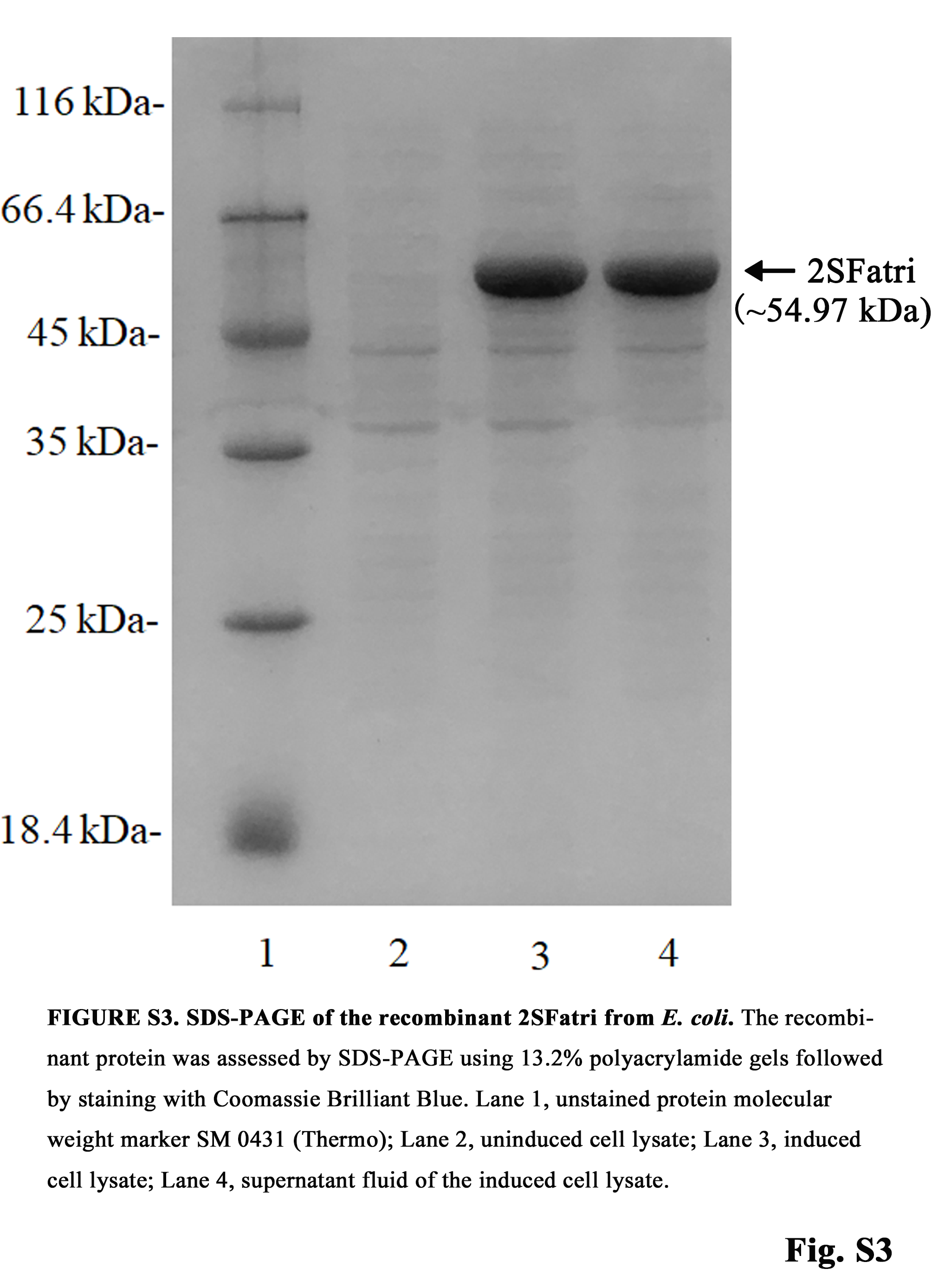

Supplement: Supplementary file 3 [file Image_3.TIF]
